# Evenness indices once again: critical analysis of properties

**DOI:** 10.1186/s40064-015-0944-4

**Published:** 2015-05-16

**Authors:** Tarald O Kvålseth

**Affiliations:** Department of Mechanical Engineering, University of Minnesota, Minneapolis, MN 55455 USA

**Keywords:** Biological evenness, Evenness indices, Value validity, Biodiversity

## Abstract

Various properties have been advocated for biological evenness indices, with some properties being clearly desirable while others appear questionable. With a focus on such properties, this paper makes a distinction between properties that are clearly necessary and those that appear to be unnecessary or even inappropriate. Based on Euclidean distances as a criterion, conditions are introduced in order for an index to provide valid, true, and realistic representations of the evenness characteristic (attribute) from species abundance distributions. Without such value-validity property, it is argued that a measure or index provides only limited information about the evenness and results in misleading interpretations and evenness comparisons and incorrect results and conclusions. Among the overabundant variety of evenness indices, each of which is typically derived by rescaling a diversity measure to the interval from 0 to 1 and thereby controlling or adjusting for the species richness, most are found to lack the value-validity property and some lack the property of strict Schur-concavity. The most popular entropy-based index reveals an especially poor performance with a substantial overstatement of the evenness characteristic or a large positive value bias. One evenness index emerges as the preferred one, satisfying all properties and conditions. This index is based directly on Euclidean distances between relevant species abundance distributions and has an intuitively meaningful interpretation in terms of relative distances between distributions. The value validity of the indices is assessed by using a recently introduced probability distribution and from the use of computer-generated distributions with randomly varying species richness and probability (proportion) components.

## Introduction

There has become an embarrassment of riches for indices used to measure the evenness or uniformity of species distributions in biology. A researcher seeking an evenness index to use in a particular study is faced with a bewildering choice. Also, there does not appear to be any general agreement as to which index is to be preferred over others. Extensive reviews of such indices and their properties have been provided by Smith and Wilson (1996) and Tuomisto (2012). Table 1 summarizes proposed evenness indices that take on values over the interval from 0 to 1 (including a new index *E*_11_).

**Table 1:** **Proposed evenness indices varying over the interval from 0 to 1 and based on the species abundance probabilities (proportions)** *p*_1_, …, *p*_*S*_ **and species richness** ***S***

**Designation**	**Formula**	**Reference**	**Notes**
*E*_1_	$H/ logS=-{\displaystyle \sum_{i=1}^S{p}_i log{p}_i/ logS}$	Pielou (1966)	
*E*_2_	(*e* ^*H*^ − 1)/(*S* − 1)	Heip (1974)	a
*E*_3_	$\Big(1-{\displaystyle \sum_{i=1}^S{p}_i^2\Big)/\left(1-1/S\right)}$	Smith and Wilson (1996)	
*E*_4_	$- log{\displaystyle \sum_{i=1}^S{p}_i^2/ logS}$	Smith and Wilson (1996)	
*E*_5_	$\Big(1/{\displaystyle \sum_{i=1}^S{p}_i^2-1\Big)/\left({e}^H-1\right)}$	Alatalo (1981)	a
*E*_6_	$\left(1-\sqrt{{\displaystyle \sum_{i=1}^S{p}_i^2}}\right)/\left(1-\sqrt{1/S}\right)$	Pielou (1969)	
*E*_7_	$\Big(1/{\displaystyle \sum_{i=1}^S{p}_i^2-1\Big)/\left(S-1\right)}$	Kvålseth (1991)	
*E*_8_	$\left({\displaystyle \sum_{i=1}^S \min \left\{{p}_i,1/S\right\}-1/S}\right)/\left(1-1/S\right)$	Bulla (1994)	
*E*_9_	$1-{\left[\left(S{\displaystyle \sum_{i=1}^S{p}_i^2-1}\right)/\left(S-1\right)\right]}^{1/2}$	Williams (1977)	b
*E*_10_	$2{\displaystyle \sum_{i=1}^S\left(i-1\right){p}_i/\left(S-1\right)}$	Solomon (1979)	c
*E*_11_	$\left(1-{\displaystyle \sum_{i=1}^S{p}_i/i}\right)/\left[1-\left(1/S\right){\displaystyle \sum_{i=1}^S1/i}\right]$	New	c

Notes: a. The *H* stands for the Shannon (1948) entropy defined for *E*_1_ and with base-e (natural) logarithms (*E*_1_ and *E*_4_ are indifferent as to which logarithm is used).

b. Engen (1979) attributed this index to F.M. Williams (1977) in an unpublished manuscript.

c. The *p*_*i*_’s are here in descending order (*p*_1_ ≥ *p*_2_ ≥ … ≥ *p*_*S*_).

Evenness indices are typically functions of some diversity measure and the number of individuals in a species sample or collection, called species richness and denoted by *S*, i.e.,

1 $$ \mathrm{Evenness}\ \mathrm{index} = f\left(\mathrm{Diversity},S\right)\in \left[0,1\right] $$

where the function *f* is generally such that the evenness index ranges in value from 0 to 1. While there is a plethora of measures of species diversity and no consensus about which measure is “best” (e.g., Magurran 2004, Ch. 4; Grassle et al. 1979; Tuomisto 2012), the present paper is confined to the measurement of species evenness with the focus on the properties of such measures or indices.

A variety of properties have been imposed on evenness indices (see, e.g., Smith and Wilson 1996; Taillie 1979; Engen 1979; Tuomisto 2012; Gosselin 2001; Ricotta 2004). Some of these appear to be entirely appropriate and generally agreed upon, while others seem to be unnecessary and even unreasonable. This paper starts off with a discussion of properties that are considered to be necessary for any acceptable evenness index, followed by comments about some dubious properties. One of the properties introduced as necessary is the *value-validity* property, which basically requires that an evenness index takes on values that are all reasonable with respect to an acceptable criterion. It is argued that this property, which nearly all proposed evenness indices lack, is necessary in order for comparisons between differences in evenness values to be valid. An index lacking this property may cause misleading results and incorrect conclusions.

The value-validity requirement of an index is based on the recently introduced *lambda distribution* (Kvålseth 2011) and a criterion involving Euclidean distances between species abundance distributions. The assessment of whether or not an index meets this requirement is then done analytically using the lambda distribution and empirically using computer-generated random species abundance distributions. It is also proved that some well-known indices are not valid since they lack the essential strict Schur-concavity property and hence do not preserve the Lorenz order.

## Properties of evenness indices

Let *E(P*_*s*_*)*, or simply *E*, represent the value of a generic evenness index for the species abundance distribution *P*_*S*_ = (*p*_1_, …, *p*_*S*_) where *p*_*i*_ is the probability (proportion) of the *i* th species, with *p*_*i*_ ≥ 0 for *i* = 1, …, *S* and ${\displaystyle \sum_{i=1}^S{p}_i=1}$. If each *p*_*i*_ is based on a sample or collection as opposed to unknown population probabilities, then p_i_ = *n*_*i*_/*N* where *n*_*i*_ is the frequency of individuals in species *i*, *i* = 1, …, *S*, and $N={\displaystyle \sum_{i=1}^S{n}_i}$ is the sample size. The extreme distributions are then

2 $$ {P}_s^1=\left(\frac{1}{S},\dots, \frac{1}{S}\right),{P}_s^0=\left(1,\kern1em 0,\dots, 0\right) $$

for which *E* is expected to take on its extremal values for any given species richness *S*. In the case of a censused community or collection of species, the most uneven species abundance distribution becomes (1 − (*S* − 1)/*N*, 1/*N*, …, 1/*N*) and the most even distribution equals ${P}_S^1$ in (2) only when *N/S* is an integer. However, when the sample size *N* is large, these two extreme distributions become effectively equivalent to those in (2).

Based on this notation, the various potential properties of an evenness index *E* will be discussed, starting with an outline and explanation of those properties that are suggested as being indeed necessary. Subsequent sections of this paper will discuss the *value-validity* property necessary for making appropriate comparisons and interpretations of *E*-values.

### Necessary properties

(P1) *Continuity*: *E* is a continuous function of each of the *p*_*i*_, *i* = 1, …, *S*, for any given *S*.

(P2) *Symmetry*: *E* is (permutation) symmetric in *p*_1_, …, *p*_*S*_ so that the value of *E* is invariant with respect to all permutations of the *p*_*i*_’s.

(P3) *Normalization*: *E* takes on values between 0 and 1.

(P4) *Schur-concavity*: *E* is strictly Schur-concave. In order to explain this property, the concept of majorization needs to be first defined. The rather vague notion that the components of *P*_*S*_ = (*p*_1_, …, *p*_*S*_) are “less spread out”, “more nearly equal”, or “more even” than are the components of the distribution *Q*_*S*_ = (*q*_1_, …, *q*_*S*_) can be expressed as “*P*_*S*_ being majorized by *Q*_*S*_” and denoted by *P*_*s*_ ≺ *Q*_*S*_. Thus, if the *p*_*i*_ (*i* = 1, …, *S*) are ordered such that

3 $$ {p}_1\ge {p}_2\ge \dots \ge {p}_S $$

with *Q*_*S*_ being similarly ordered, then, by definition,

4 $$ {P}_S\prec {Q}_S\kern0.5em \mathrm{if}\kern0.5em {\displaystyle \sum_{j=1}^i{p}_j\le {\displaystyle \sum_{j=1}^i{q}_j}},\kern0.5em i=1,\dots, S-1 $$

and with ${\displaystyle \sum_{i=1}^S{p}_i={\displaystyle \sum_{i=1}^S{q}_i=1}}$. Then, by definition, *E* is strictly Schur-concave if

5 $$ {P}_S\prec {Q}_S\kern0.5em \mathrm{implies}\kern0.5em E\left({P}_S\right)>E\left({Q}_S\right) $$

and *P*_*S*_ is not a permutation of *Q*_*S*_ (Marshall et al. 2011, pp. 8, 80).

As a simple numerical example, consider the two distributions *P*_4_ = (0.40, 0.30, 0.20, 0.10) and *Q*_4_ = (0.70, 0.15, 0.10, 0.05) where the components have been ordered as in (3). It is readily apparent from (4) that *P*_4_ is majorized by *Q*_4_, i.e., *P*_4_ ≺ *Q*_4_ Then, since the evenness index *E* is required to be strictly Schur-concave, *E*(*P*_4_) > *E*(*Q*_4_) from (5). Clearly, the components of *P*_4_ are “more evenly distributed” or “more nearly equal” than those of *Q*_4_ and the evenness based on *P*_4_ should be greater than that based on *Q*_4_. This is precisely what Property P4 requires.

(P5) *Value Validity*: *E* takes on values that are all valid representations of the true extent of the species evenness characteristic.

This last property, which is considered necessary for making appropriate evenness difference comparisons, is explained in detail in subsequent sections of the paper.

### Comment on property P1

The continuity requirement ensures that, for any given or fixed *S*, small changes in some of the *p*_*i*_ ’s cause only a small change in the value of *E*. However, since *E* may be a function of both the *p*_*i*_(*i* = 1, …, *S*) and *S*, a change in *S* and hence changes in the *p*_*i*_’s will necessarily result in a discontinuous change in *E*. In fact, if *S* is very small, the addition of one more species may produce a substantial jump in the value of *E*. Such discontinuity with varying *S* has been discussed by, for instance, Routledge (1983), Jost (2010), and Ricotta (2004).

### Other implications from property P4

The strict Schur-concavity Property P4 of *E* has some important implications. Since, for any *P*_*s*_ = (*p*_1_, …, *p*_*s*_), it is readily apparent from the definition in (4) that ${P}_S^1\prec {P}_S\prec {P}_S^0$ for the ${P}_S^1$ and ${P}_S^0$ in (2), it then follows from (5) that

6 $$ E\left({P}_S^0\right)\le E\left({P}_S\right)\le E\left({P}_S^1\right) $$

with the inequalities being strict if *P*_*S*_ is not a permutation of ${P}_S^0$ or ${P}_S^1$. This can be expressed as the following sub-properties:

(P4a) For any given species richness *S*, *E* attains its minimum and maximum values for the species abundance distributions ${P}_S^0$ and ${P}_S^1$ in (2), respectively.

Another consequence of Property P4 is that *E* satisfies the *principle of transfers*, which, within the context of income distributions, was introduced by Dalton (1920). That is, if *p*_*i*_ < *p*_*j*_ in the species abundance distribution *P*_*S*_ = (*p*_1_, …, *p*_*S*_) and if an amount *δ* is transferred from *p*_*j*_ to *p*_*i*_, with *δ* < *p*_*j*_ − *p*_*i*_, then the value of *E* increases. This can be seen to follow from (3)-(5). See also Marshall et al. (2011), pp. 6–8. That is, a sub-property of *E* is as follows:

(P4b) *E* satisfies the transfer property. As a simple example, consider the four-species distribution *Q*_4_ = (0.30, 0.15, 0.50, 0.05) with *δ* = 0.20 being transferred from *p*_3_ to *p*_4_ to produce the distribution *P*_4_ = (0.30, 0.15, 0.30, 0.25). It is then clear from (3)-(4) that *P*_4_ is majorized by *Q*_4_, so that, from (5) and the strict Schur-concavity of *E*, *E*(*P*_4_) > *E*(*Q*_4_).

A third consequence of Property P4 may be stated in terms of Lorenz curves as follows (see Marshall et al. 2011, pp. 5–6, 713–715):

(P4c) *E* preserves the Lorenz order. As an explanation, the Lorenz curve for a distribution *P*_*S*_ = (*p*_1_, …, *p*_*S*_) is typically based on the cumulative probabilities ${F}_{(i)}={\displaystyle \sum_{j=1}^i{p}_{(j)}}$ after the *p*_*i*_’s have been ordered such that *p*_(1)_ ≤ *p*_(2)_ ≤ … ≤ *p*_(*S*)_. The Lorenz curve (after Lorenz 1905) is then obtained by joining the consecutive points (*i*/*S*, *F*_(*i*)_) for *i* = 0, 1, …, *S* and *F*_(0)_ = 0 by successive line segments to connect the origin (0, 0) with the point (1, 1). If the Lorenz curve for *P*_*S*_ is nowhere below and does not coincide everywhere with the Lorenz curve for the distribution *Q*_*S*_ = (*q*_1_, …, *q*_*S*_), this is equivalent to the majorization in (3)-(4) so that, from (5), *E*(*P*_*S*_) > *E*(*Q*_*S*_). The Lorenz curve for the uniform or completely even distribution ${P}_S^1$ in (2) is the straight line between the two points (0, 0) and (1, 1) so that the value *E(P*_*S*_*)* increases as the Lorenz curve for *P*_*S*_ gets closer to this straight line.

### Introductory comments on value validity

The motivation behind the need for an evenness index to have value validity can perhaps be best illustrated by means of some simple numerical examples. Consider, for instance, the species abundance distributions *P*_2_ = (0.75, 0.25) and *Q*_5_ = (0.60, 0.10, 0.10, 0.10, 0.10) and the Pielou index *E*_1_ in Table 1 for which *E*_1_(*P*_2_) = 0.81 and *E*_1_(*Q*_5_) = 0.76. Both of these results would seem to indicate a “high” degree of evenness, with *P*_2_ indicating a more even distribution than *Q*_5_ of about 7%. However, the components of each of these two distributions are equally far from the corresponding components of the respective pairs of extreme distributions ${P}_2^0$ and ${P}_2^1$ and ${Q}_5^0$ and ${Q}_5^1$ defined in (2) so that the only reasonable evenness values should be 0.50 for both *P*_2_ and *Q*_5_. Furthermore, if, say, $\sqrt{E_1}$ or more generally ${E}_1^{\alpha }\ \left(\alpha >0\right)$ were to be considered as alternative indices, which can be shown to have the same Properties P1-P4 as *E*_1_, the results could be even more unreasonable such as $\sqrt{E_1\left({Q}_5\right)}=0.87$.

Of course, the purpose of using an evenness index *E* is to compare the degree or extent of evenness of different species abundance distributions. Thus, in simplified notation, if *e*_1_, *e*_2_, … denote values of the index *E* for different species distributions, the various comparisons of potential interest may be defined as follows:

7a $$ Size\ (order)\  comparisons:\kern0.5em {e}_1>{e}_2 $$

7b $$ Difference\  comparisons:\kern0.5em {e}_1-{e}_2>{e}_3-{e}_4 $$

7c $$ Proportional\  difference\  comparisons:{e}_1-{e}_2=c\left({e}_3-{e}_4\right) $$

where *c* is some constant. Even the above interpretation of *E*_1_(0.75, 0.25) = 0.81 as indicating a “high” degree of evenness is basically a difference comparison as in (7b), with 0.81 − 0 > > 1 − 0.81, i.e., the 0.81 value is much further from the minimum *E*_1_ -value of 0 than it is from the maximum *E*_1_ -value of 1. Similarly, the above statement that *E*_1_(0.75, 0.25) = 0.81 exceeds *E*_1_(0.60, 0.10, …, 0.10) by about 7% is equivalent to *e*_1_ = 0.81, *e*_2_ = *e*_3_ = 0.76, *e*_4_ = 0, and *c* = 0.07 in (7c).

When making such comparisons, it is essential that they are not limited to the evenness index itself, but that they provide true representations of the attribute (characteristic) being measured. Otherwise, the results obtained may be invalid and misleading, which is not uncommon in the published literature. What is needed of the index *E* is that it takes on values throughout its range that are all accurate, true, or valid representations of the evenness attribute. That is, *E* has to have *value validity* (Property P5)*.* In measurement theory, the term *validity* of a measurement procedure means that it does in fact measure what it purports to measure. While several different types of validity have been defined (see, e.g., Hand 2004, pp. 129–134), *value validity* as used here applies specifically to the requirement that all potential numerical values of a measure have to be appropriate or reasonable with respect to some generally acceptable criterion as explained in the next subsection of this paper.

The importance of a concept such as value validity does not appear to have been the subject of rigorous attention in the numerous publications involving evenness measurement and indices. Some have commented that certain evenness indices “tend to strongly overestimate evenness” or “give unreasonably high values of evenness” (Bulla 1994, pp. 167, 169). The results from computer-generated combinations of species frequencies by Fager (1972) implied that indices such as Pielou’s *E*_1_ and Smith and Wilson’s *E*_3_ in Table 1 tended to overstate evenness and had “strongly skewed distributions…” indicating that those indices “would give poor discrimination over much of the range of samples (p. 301).” Molinari (1989) did consider the importance of reasonable intermediate values of an evenness index, focusing on the case of two-species communities, and Smith and Wilson (1996) as well as Jost (2010) emphasized the need to consider intuitively reasonable intermediate index values. However, no specific value-validity conditions appear to have been formulated.

### Conditions for value validity

In order to establish value-validity conditions on *E* (Property P5), it will be convenient to use the following *lambda distribution* recently introduced by this author (Kvålseth 2011, 2014):

8 $$ {P}_S^{\lambda }=\left(1-\lambda +\frac{\lambda }{S},\frac{\lambda }{S},\dots, \frac{\lambda }{S}\right),0\le \lambda \le 1 $$

where *λ* is a parameter and *S* denotes the species richness. The *λ* is basically an evenness or uniformity parameter with *λ* = 1 for the completely even (uniform) distribution ${P}_S^1$ in (2) and *λ* = 0 for the degenerate distribution ${P}_S^0$ in (2). Note also that each component of ${P}_S^{\lambda }$ can be considered the weighted mean of components of ${P}_S^0$ and ${P}_S^1$, i.e.,

9 $$ {P}_S^{\lambda }=\lambda {P}_S^1+\left(1-\lambda \right){P}_S^0 $$

Since the strict Schur-concavity of *E* ensures that the inequality in (6) holds for all distributions *P*_*S*_ = (*p*_1_, …, *p*_*S*_), it also holds for ${P}_S^{\lambda }$ and all *S* and *λ*. Therefore, for any given *P*_*S*_, there exists one, and only one, value of *λ* such that

10 $$ E\left({P}_S\right)=E\left({P}_S^{\lambda}\right)\kern0.5em \mathrm{f}\mathrm{o}\mathrm{r}\ \mathrm{any}\ \mathrm{given}\kern0.5em {P}_S\kern0.5em \mathrm{and}\ \mathrm{o}\mathrm{ne}\ \mathrm{unique}\kern0.5em \lambda \hbox{-} \mathrm{value} $$

Of course, for any given *S*, there may be any number of different *P*_*S*_ -distributions for which the value of *E* would be the same and hence for which (10) would apply. Because of (10), the value validity of *E* and appropriate conditions can be considered in terms of the ${P}_S^{\lambda }$ -distribution in (8).

Consider now that each distribution *P*_*S*_ is a point or vector in *S*-dimensional Euclidean space with Cartesian coordinates *p*_1_, …, *p*_*S*_. The Euclidean distance between the points *P*_*S*_ and *Q*_*S*_ is given by $d\left({P}_S,{Q}_S\right)={\left[{\displaystyle \sum_{i=1}^S{\left({p}_i-{q}_i\right)}^2}\right]}^{1/2}$. A most logical requirement for value validity based on Euclidean distances may then be formulated as the following equality between distance ratios:

11 $$ \frac{\left|E\left({P}_S^{\lambda}\right)-E\left({P}_S^1\right)\right|}{\left|E\left({P}_S^0\right)-E\left({P}_S^1\right)\right|}=\frac{d\left({P}_S^{\lambda },{P}_S^1\right)}{d\left({P}_S^0,{P}_S^1\right)} $$

for the ${P}_S^0,\kern0.5em {P}_S^1,\kern0.5em {P}_S^{\lambda }$ defined in (2) and (8). Since the right-hand side ratio in (11) can be seen to equal 1 − *λ* and from the Schur-concavity Property P4, specifically (6), the relationship in (11) can equivalently be expressed as

12 $$ E\left({P}_S^{\lambda}\right)=\lambda E\left({P}_S^1\right)+\left(1-\lambda \right)E\left({P}_S^0\right),\kern0.5em 0\le \lambda \le 1 $$

13 $$ =\lambda \kern0.5em \mathrm{f}\mathrm{o}\mathrm{r}\kern0.5em E\left({P}_S^1\right)=1\kern0.5em \mathrm{and}\kern0.5em E\left({P}_S^0\right)=0 $$

The distance-based condition in (12) is also a logical implication from (9). That is, the evenness value in (12) consists of a weighted mean equivalent to that of the underlying species distributions in (9).

The condition in (11) and hence in (12)-(13) applies specifically to the lambda distribution ${P}_S^{\lambda }$ in (8). However, because of (10), it seems entirely reasonable to also require that (11) should generally provide a good (close) approximation when ${P}_S^{\lambda }$ is replaced by any species distribution *P*_*S*_ = (*p*_1_, …, *p*_*S*_). Therefore, for any distribution *P*_*S*_, the approximation corresponding to (13) becomes

14 $$ E\left({P}_S\right)\approx 1-\frac{d\left({P}_S,{P}_S^1\right)}{d\left({P}_S^0,{P}_S^1\right)} $$

so that, with $E\left({P}_S^0\right)=0$ and $E\left({P}_S^1\right)=1$, (13) and (14) become conditions for value validity of the evenness index *E*.

## Further property comments

### Distance criterion

The Euclidean distance is the metric being used as the basic criterion for the value-validity conditions in (11)-(14). Other metric distance functions could have been considered such as alternative members of the Minkowski family ${d}_{\alpha}\left({P}_S,{Q}_S\right)={\left[{\displaystyle \sum_{i=1}^S{\left|{p}_i-{q}_i\right|}^{\alpha }}\right]}^{{}^{1/\alpha }}$ for parameter *α* ≥ 1, with *α* = 2 being the Euclidean metric and *α* = 1 being the so-called city-block or Manhattan metric (e.g., Upton and Cook 2008, pp. 118–119). While it is easily seen that, if any member of this distance family is used in (11), the expressions in (12)-(13) would still apply, but the right-hand side of (14) would generally differ for different values of *α*. The Euclidean distance is used here since it is the standard or ordinary measure of distance used in mathematics and the various sciences. The use of any other alternative distance metric or function would seem to require some particular justification or explanation.

Also, it is of importance to note that the right-hand side of (14) based on the Euclidean distance is strictly Schur-concave whereas, had it been based on the city-block distance, the corresponding expression would have been Schur-concave, but not strictly Schur-concave. This follows from the fact that (a) the Euclidean distance between *P*_*S*_ and ${P}_S^1$ is strictly Schur-convex while the city-block distance is Schur-convex, but not strictly so (Kvålseth 2011), and (b) the right-hand side of (14) is a strictly decreasing function of those two alternative distance metrics (Marshall et al. 2011, pp. 88–91, 138–139).

### Bounds on *E*

For a censused community with species abundance frequencies *n*_1_, …, *n*_*S*_ and total number of individuals $N={\displaystyle \sum_{i=1}^S{n}_i}$, the most uneven species abundance distribution becomes ${P}_S^{0+}=\left(1-\left(S-1\right)/N,1/N,\dots, 1/N\right)$ and the most even distribution ${P}_S^{1-}$ equals ${P}_S^1$ in (2) only if *N*/*S* is an integer. For these extremal distributions, none of the evenness indices in Table 1 can attain the lower and upper bounds of 0 and 1 since ${E}_i\left({P}_S^{0+}\right)>0$ and ${E}_i\left({P}_S^{1-}\right)<1$. Of course, when *N* is large, ${P}_S^{0+}$ and ${P}_S^{1-}$ become effectively equal to the distributions in (2) and ${E}_i\left({P}_S^{0+}\right)\approx 0$ and ${E}_i\left({P}_S^{1-}\right)\approx 1$.

As a simple example, consider *S* = 5 and *N* = 98. Then, from the above definition, ${P}_5^{0+}=\left(1-4/98,\kern0.5em 1/98,\dots, \kern0.5em 1/98\right)$ for the frequencies *n*_*i*_ = 94, 1, …, 1 and ${P}_5^{1-}=\left(20/98,\ 20/98,\ 20/98,\ 19/98,\ 19/98\right)$. For, say, the Williams’ *E*_9_ in Table 1, the values for these two extreme distributions are found to be ${E}_9\left({P}_5^{0+}\right)=0.05$ and ${E}_9\left({P}_5^{1-}\right)=0.99$ as compared to ${E}_9\left({P}_5^0\right)=0$ and ${E}_9\left({P}_5^1\right)=1$ for the ${P}_5^0$ and ${P}_5^1$ in (2).

Some (e.g., Fager 1972) have suggested that an evenness index should be able to attain the values 0 and 1 for any *S* and *N* while others do not impose such requirement as long as an index takes on values near 0 and 1 when the species distribution is as uneven or even as possible for any given *S* and *N* (e.g., Smith and Wilson 1996). Of course, an index *E* with $E\left({P}_S^{0+}\right)>0$ and $E\left({P}_S^{1-}\right)<1$ can easily be rescaled to the interval [0,1] by defining

15 $$ {E}^{\bullet}\left({P}_S\right)=\frac{E\left({P}_S\right)-E\left({P}_S^{0+}\right)}{E\left({P}_S^{1-}\right)-E\left({P}_S^{0+}\right)} $$

In the above example, with *S* = 5 and *N* = 98, consider *n*_*i*_ = 20, 5, 60, 10, 3 for which the value of *E*_9_ in Table 1 becomes *E*_9_(*P*_5_) = 0.46 whereas, from (15), ${E}_9^{\bullet}\left({P}_5\right)=\left(0.46-0.05\right)/\left(0.99-0.05\right)=0.44$.

However, there seems to be no particular basis for imposing such a rescaling requirement on an evenness index. In fact, on intuitive grounds, it seems unreasonable. To suggest that the above distribution ${P}_5^{1-}=\left(20/98,\kern0.5em 20/98,\kern0.5em 20/98,\kern0.5em 19/98,\kern0.5em 19/98\right)$ indicates complete evenness is simply not appropriate. Rather, this ${P}_S^{1-}$ is as even as it can be when *S* = 5 and *N* = 98. Similarly, the above distribution ${P}_5^{0+}=\left(94/98,1/98,\dots, 1/98\right)$ is as uneven as possible for given *S* = 5 and *N* = 98, but it should not be considered to imply perfect unevenness. The above value, ${E}_9\left({P}_5^{0+}\right)=0.05$ rather than zero, would seem to provide a reasonable representation of the evenness.

### Independence of *S*

It has been argued strongly by some that an evenness index *E* should be independent of the species richness *S* (e.g., Peet 1975; Hill 1973; Heip et al. 1998). Such a requirement would eliminate all indices in Table 1 for an insufficient reason. It is probably inevitable that any *E* with the above Properties P1-P5 will to some extent depend on *S*. This dependence has also been discussed more recently by Gosselin (2006) and Jost (2010).

It can certainly be argued that the dependence of *E* on *S* is not by itself an undesirable property. The purpose of an evenness index *E* is to measure how evenly (uniformly) the relative abundances *p*_*i*_(*i* = 1, …, *S*) are distributed across the *S* different species, irrespective of the value of *S*. The reason for normalizing (rescaling) an evenness index such that the values of *E* all fall within the interval from 0 to 1 (or nearly so) is simply to be able to properly compare values of *E* for species distributions with differing *S* -values. That is, the normalized *E* controls or adjusts for *S*. Without such control or adjustment of *S*, an evenness index would be a confounded representation of species richness *S* and the form of the species distribution. For such an index, one would not be able to tell whether different index values were due to differences in *S* -values or differences in the form of species distributions or both. An evenness index *E* ∈ [0, 1] is only supposed to measure the form of the species distribution, thereby requiring the control (adjustment) for *S*, and thus making *E* a function of both *p*_*i*_, …, *p*_*S*_ and *S*.

In statistical analysis of categorical data, an evenness index such as *E*_3_ in Table 1 has been used as a measure of variation and referred to as the *index of qualitative variation (IQV)* (e.g., Mueller and Schuessler 1977, pp. 175–181; Reynolds 1984, pp. 61–64). For such measurement, the fact that the normalized *E*_3_ ∈ [0, 1] depends on the number of categories *S* does not appear to be much of an issue of concern, while papers on biological evenness seem to have made the dependence on *S* into an unnecessary issue. Just as it is for a measure of qualitative variation, inclusion of *S* into the formulation of *E* ∈ [0, 1], besides the ease of interpretation, is precisely for the purpose of obtaining an evenness index that can be used to compare the evenness of different species distributions of differing *S*.

One type of situation in which there is reason for genuine concern about *E* depending on species richness is when the relative species frequencies *p*_*i*_ = *n*_*i*_/*N* (*i* = 1, …, *S*) and sample size $N={\displaystyle \sum_{i=1}^S{n}_i}$ are based on random observations from a population of species and when the computed (sample) value *E*(*n*_1_/*N*, …, *n*_*S*_/*N*) is used to make statistical inferences about the corresponding unknown population index *E*_*p*_. Such statistical inferences would include (a) the use of the sample value of *E* as an appropriate estimate of *E*_*p*_ and (b) the construction of confidence intervals for *E*_*p*_. However, the necessary conditions for making such statistical inferences are rarely met in biological studies. It is typical for such sampling data that neither the total number of species *S*_*p*_ in the defined population nor all the specific types of species are known, with *S* < *S*_*p*_ and *S* depending to some extent on the sample size *N*. It is then most prudent to confine the evenness measurement to the sample itself.

### Replication principle

A measurement property that has also been referred to as the “replication property” or “replication principle” was introduced by Dalton (1920) for the measurement of economic inequality. Thus, if (*x*_1_, …, *x*_*n*_) denotes the set of incomes of *n* individuals and if another set of identical incomes for *n* other individuals are combined to produce the set of incomes (*x*_1_, *x*_1_, *x*_2_, *x*_2_, …, *x*_*n*_, *x*_*n*_) for 2*n* individuals, then a measure of income inequality should have the same value for (*x*_1_, *x*_1_, …, *x*_*n*_, *x*_*n*_) as for (*x*_1_, …, *x*_*n*_). Any number of such replications should produce the same result. Dalton (1920) called this the “principle of proportionate additions to persons”, while others (e.g., Cowell 2011, pp. 63–64) have referred to it as the “principle of population”. The term “replication” property or principle, as also used for biological evenness measures (e.g., Taillie 1979; Tuomisto 2012), would seem to be most descriptive and will be used here.

However, it may perhaps be questionable if such a replication property should be considered necessary or even desirable, especially when the underlying data are categorical such as biological species frequencies (counts) as opposed to quatitative data such as personal incomes. Even when measuring income inequality, some feel that the desirability of this property is debatable and not self-evident (Cowell 2011, pp. 63–64), while others do not even mention this property when discussing inequality measures (Bellù and Liberati 2006; Marshall et al. 2011, Sec 13F).

For quantitive data, it does at least make realistic sense to consider a replication (*x*_1_, *x*_1_, …, *x*_*n*_, *x*_*n*_). However, for categorical data such as a set of biological species abundance frequencies (counts) (*n*_1_, *n*_2_, …, *n*_*S*_), a replication (*n*_1_, *n*_1_, *n*_2_, *n*_2_, …, *n*_*S*_, *n*_*S*_) would seem to be an unrealistic and intuitively meaningless concept. Each *n*_*i*_ has to be identified with a specific species (or category) within a set of mutually exclusive and exhaustive set of species of size *S*. If, say, (*n*_1_, …, *n*_*S*_) are the frequencies for a set of *S* different types of apple trees in one geographic area and if exactly the same frequencies are observed for the same set of *S* apple trees in an adjacent area, it would make no sense to determine an evenness value based on the data (*n*_1_, *n*_1_, …, *n*_*S*_, *n*_*S*_) for the two combined areas. Such determination should be based on (2*n*_1_, …, 2*n*_*S*_). Otherwise, one would be “comparing apples and oranges”.

One situation in which it does make sense to consider replication would be when a set of *S* different species with frequencies (*n*_1_, …, *n*_*S*_) are split into males and females, resulting in the species-sex frequencies (*n*_1_/2, *n*_1_/2, …, *n*_*S*_/2, *n*_*S*_/2). The *S* “species” categories have been split into 2*S* “species-sex” categories. These are two different sets of categories for which there would seem to be no basis for requiring that the evenness should be the same.

Jost (2010) has also argued against the requirement that an evenness index should possess the replication property. In fact, he argues that replication invariance would be an undesirable property for an evenness index *E* ∈ [0, 1].

Any proposed condition such as the one involving replication also has to make some intuitive sense when simply looking at the data, both in terms of frequencies *n*_*i*_ and proportions (relative frequencies) *p*_*i*_ = *n*_*i*_/*N* for *i* = 1, …, *S* and $N={\displaystyle \sum_{i=1}^S{n}_i}$. For instance, consider two sets of data: frequencies (80, 20) or proportions *P*_2_ = (0.80, 0.20) versus the replicated (80, 80, 20, 20) or *Q*_4_ = (0.40, 0.40,0.10, 0.10). It would be hard to justify the proposition that both data sets reveal the same degree of evenness, especially when considering the *P*_2_ versus the *Q*_4_. Clearly, *Q*_4_ indicates greater evenness than *P*_2_ since the components of *Q*_4_ differ less than those of *P*_2_.

As an example of a replication-invariant evenness index producing such intuitively unreasonable values, consider

16 $$ {E}_T=\frac{1}{S}\left(2{\displaystyle \sum_{i=1}^Si{p}_i-1}\right)\in \left[\frac{1}{S},1\right] $$

where *p*_1_, …, *p*_*S*_ are ordered as in (3) (Taillie 1979, p. 56). This *E*_*T*_ represents a slight modification of Solomon’s *E*_10_ in Table 1. Because of its lower bound 1/*S*, an interpretation such as *E*_*T*_(1, 0) = 0.50 showing more than twice the evenness of *E*_*T*_(1, 0, 0, 0, 0) = 0.20 makes no intuitive sense. Similarly, in terms of frequencies *n*_1_, …, *n*_*S*_, with $N={\displaystyle \sum_{i=1}^S{n}_i,}$ it is found from (16) with *p*_*i*_ = *n*_*i*_/*N* (*i* = 1, … *S*) that

$$ {E}_T\left(N-S+1,1,\dots, 1\right)=\frac{1}{S}+\frac{S-1}{N} $$

with such unreasonable results as *E*_*T*_(99, 1) = 0.51 and *E*_*T*_(96, 1, 1, 1, 1) = 0.24. By comparison, from the *E*_9_ in Table 1, *E*_9_(99, 1) = 0.02 < *E*_9_(96, 1, 1, 1, 1) = 0.05, an intuitively reasonable result.

From the definition of the Lorenz curve under Property P4c, it is readily seen that the Lorenz curve of, say, *P*_2_ = (0.80, 0.20) and *Q*_4_ = (0.40, 0.40, 0.10, 0.10) coincide (one curve with two more points than the other), a result that holds for all replication situations. However, considering the preceding arguments, such a Lorenz-curve fact would not be sufficient to argue for a proposition that an evenness index should be invariant with respect to replication.

## Assessment of proposed indices

Based on the above discussion of appropriate properties for an evenness index, the validity of various proposed indices may now be assessed. All of the indices listed in Table 1 can be seen to meet the requirements for continuity, symmetry, and normalization, i.e., Properties P1-P3. However, as will be explained next, some of those indices are not strictly Schur-concave (Property P4) and most do not satisfy the conditions for value validity (Property P5).

### Schur-concavity

Alatalo’s index *E*_5_ in Table 1 is not Schur-concave. This is rather apparent since the denominator of *E*_5_ is strictly Schur-concave because (a) the entropy *H* is strictly Schur-concave and (b) a strictly increasing function of a strictly Schur-concave function is strictly Schur-concave (Marshall et al. 2011, pp. 89, 101). Also, as an example, consider the distributions *P*_3_ = (0.80, 0.10, 0.10) and *Q*_3_ = (0.80, 0.19, 0.01) for which *E*_5_(*P*_3_) = 0.58 exceeds *E*_5_(*Q*_3_) = 0.67 even though *P*_3_ is majorized by *Q*_3_ so that *E*_5_ cannot be Schur-concave (see (3)-(5)). Consequently, *E*_5_ is not a valid evenness index.

Bulla’s index *E*_8_ in Table 1 is Schur-concave, but it is not strictly Schur-concave as required by Property P4. A proof of the Schur-concavity of *E*_8_ is given below in Appendix A. The fact that this index is not strictly Schur-concave can be verified by the following counterexample: *E*_8_(0.80, 0.15, 0.05) = *E*_8_(0.80, 0.10, 0.10) = 0.30 even though (0.80, 0.10,0.10) is majorized by (0.80,0.15,0.05) as can be seen from (3)-(4). Consequently, from (5), *E*_8_ cannot be strictly Schur-concave.

Among other proposed evenness indices, Smith and Wilson (1996) introduced one as a strictly decreasing function of the variance *V* of the logged frequencies, i.e., $V={\displaystyle \sum_{i=1}^S{\left[ log{n}_i-{\displaystyle \sum_{i=1}^S\left( log{n}_i\right)/S}\right]}^2/S}$. However, since *V* is not Schur-convex and a decreasing function of *V* is therefore not Schur-concave (Marshall et al. 2011, pp. 89, 561), the index proposed by Smith and Wilson (1996) is not Schur-concave and is therefore not a valid evenness index.

Hill (1973) proposed a family of evenness indices as

17 $$ {E}_{H\alpha \beta }=\frac{N_{\alpha }}{N_{\beta }},\ {N}_{\alpha }={\left({\displaystyle \sum_{i=1}^S{p}_i^{\alpha }}\right)}^{1/\left(1-\alpha \right)},\ {N}_{\beta }={\left({\displaystyle \sum_{i=1}^S{p}_i^{\beta }}\right)}^{1/\left(1-\beta \right)} $$

with no restriction on the parameters *α* and *β*. However, in order for a member of this family to be strictly Schur-concave, it is necessary that *α* > 0 and *β* < 0. These parameter ranges do not include the specific member *E*_*H*2,1_ for *α* = 2 and *β* = 1 in (17) mentioned by Hill (1973) and of which the *E*_5_ in Table 1 is a slight modification. Also, *E*_*Hαβ*_ in (17) is undefined for *β* < 0 unless it is assumed that all *p*_*i*_ are positive. A proof of these parameter restrictions is given below in Appendix B.

Hill (1997) also proposed the evenness index

$$ {\left(\frac{N_3}{N_2}\right)}^2=\frac{{\left({\displaystyle \sum_{i=1}^S{p}_i^2}\right)}^2}{{\displaystyle \sum_{i=1}^S{p}_i^3}} $$

where *N*_2_ and *N*_3_ are defined from (17). This index is replication invariant and relatively independent of *S*, but, as can be inferred from the preceding paragraph, it cannot be Schur-concave since it is a ratio of Schur-convex functions. The fact that this index is not Schur-concave and therefore not a valid evenness index is also evident from, for instance, the two distributions (0.6, 0.4) and (0.99, 0.01) for which this Hill’s index takes on the respective values 0.97 and 0.99. This is clearly an absurd result since the components of (0.6, 0.4) are more even than those of (0.99, 0.01), i.e., (0.6, 0.4) is majorized by (0.99, 0.01) (see (3)-(4)).

Ricotta (2004) made an interesting attempt to formulate an evenness index by using a fuzzy set theory approach. With the *p*_*i*_’s ordered as in (3), this index is defined as

$$ {E}_R=1-{\displaystyle \sum_{i=1}^S\left(\frac{p_i-{p}_{i+1}}{i}\right)} $$

where *p*_*S* + 1_ = 0. However, this index is not Schur-concave (nor is it Schur-convex) and is therefore not a valid evenness index. This fact can be proved by using a counterexample such as the two species distributions *P*_3_ =(0.6, 0.2, 0.2) and *Q*_3_ = (0.6, 0.3, 0.1) where *Q*_3_ is derived from *P*_3_ by means of the transfer of 0.1 from *p*_3_ to *p*_2_. From the definition in (4), it is clear that *P*_3_ is majorized by *Q*_3_, but *E*_*R*_(*P*_3_) = 0.53 < *E*_*R*_(*Q*_3_) = 0.57 so that, from (5), *E*_*R*_ is not Schur-concave.

### Value validity (condition (13))

Of the evenness indices identified so far in this paper, most of them lack the value validity property as defined and discussed above. Those indices cannot therefore be used to make valid difference comparisons as in (7b)-(7c) and their numerical values cannot be used as valid indicators of the true extent of evenness from a data set. Only “larger than” comparisons as in (7a) may be valid for those indices. Among the indices defined in Table 1, only *E*_8_, *E*_9_, *E*_10_, and *E*_11_ can be seen to meet the condition in (13) for value validity. However, as proved above, Bulla’s *E*_8_, while being Schur-concave, lacks the strict Schur-concavity property. As a demonstration of such lack of value validity, consider the results in Table 2 giving the values of the measures in Table 1 for the lambda distribution ${P}_S^{\lambda }$ in (8) with some different values of *λ* and *S*.

**Table 2:** **Values of the evenness indices in Table** **1** **for the lambda distribution** ${P}_S^{\lambda }$ **in (8) with different** ***λ*** **and** ***S*** **values**

***S***	***λ***	***E***_**1**_	***E***_**2**_	***E***_**3**_	***E***_**4**_	***E***_**5**_	***E***_**6**_	***E***_**7**_	***E***_**8**_	***E***_**9**_	***E***_**10**_	***E***_**11**_
2	0.2	0.47	0.38	0.36	0.29	0.57	0.61	0.22	0.20	0.20	0.20	0.20
2	0.5	0.81	0.75	0.75	0.68	0.79	0.72	0.60	0.50	0.50	0.50	0.50
2	0.8	0.97	0.96	0.96	0.94	0.96	0.95	0.92	0.80	0.80	0.80	0.80
5	0.2	0.41	0.23	0.36	0.21	0.43	0.28	0.10	0.20	0.20	0.20	0.20
5	0.5	0.76	0.60	0.75	0.57	0.62	0.66	0.38	0.50	0.50	0.50	0.50
5	0.8	0.96	0.92	0.96	0.91	0.90	0.94	0.83	0.80	0.80	0.80	0.80
10	0.2	0.38	0.15	0.36	0.17	0.35	0.26	0.05	0.20	0.20	0.20	0.20
10	0.5	0.73	0.48	0.75	0.49	0.48	0.63	0.23	0.50	0.50	0.50	0.50
10	0.8	0.94	0.87	0.96	0.87	0.81	0.92	0.71	0.80	0.80	0.80	0.80
20	0.2	0.35	0.10	0.36	0.14	0.28	0.24	0.03	0.20	0.20	0.20	0.20
20	0.5	0.70	0.37	0.75	0.42	0.35	0.60	0.13	0.50	0.50	0.50	0.50
20	0.8	0.93	0.80	0.96	0.81	0.68	0.91	0.55	0.80	0.80	0.80	0.80
30	0.2	0.34	0.07	0.36	0.13	0.25	0.24	0.02	0.20	0.20	0.20	0.20
30	0.5	0.68	0.32	0.75	0.38	0.29	0.58	0.09	0.50	0.50	0.50	0.50
30	0.8	0.92	0.76	0.96	0.77	0.58	0.89	0.44	0.80	0.80	0.80	0.80

It is clearly indicated by these results that, except for *E*_8_, …, *E*_11_, the various indices violate the condition in (13) that $E\left({P}_S^{\lambda}\right)=\lambda$, some more so than others. For some of the indices, their values for the distribution ${P}_S^{\lambda }$ exceed the *λ* -values, i.e., they overstate the extent of evenness, while such bias is reversed for other indices, depending upon the values of *λ* and *S*. Although these results are based on the specific distribution ${P}_S^{\lambda }$ in (8), they have definite implications for species distributions *P*_*S*_ = (*p*_1_, …, *p*_*S*_) in general because of the relationship in (10).

It is then apparent from Table 2 together with (10) that the general tendency for each of the *E*_1_, …, *E*_6_ is to overstate the degree of evenness, especially when the species richness *S* is small. The indices *E*_1_, *E*_3_, and *E*_6_ appear to consistently overstate the evenness. In terms of the *value bias* of an evenness index *E* (*VBE*) (as distinct from statistical bias of an estimator) defined as

18 $$ VBE\left({P}_S^{\lambda}\right)=E\left({P}_S^{\lambda}\right)-\lambda $$

*E*_1_, *E*_3_, and *E*_6_ appear to have such a consistent positive bias for all ${P}_S^{\lambda }$ and hence generally for all *P*_*S*_ = (*p*_1_, …, *p*_*S*_) from (10). Interestingly, such positive bias for *E*_3_ does not depend explicitly on *S* since, from (18) and the expression for *E*_3_ in Table 1, this bias is found to be $VB{E}_3\left({P}_S^{\lambda}\right)=\lambda \left(2-\lambda \right)-\lambda =\lambda \left(1-\lambda \right)$, which also is clearly maximal for *λ* = 0.5. Note also in Table 2 for *S* > 2 the extremely large negative value bias for the index *E*_7_ proposed by this author (Kvålseth 1991), making it entirely invalid for the difference comparisons in (7b)-(7c).

For the evenness indices *E*_9_, *E*_10_, and *E*_11_, which all have Properties P1-P4 and which meet the condition in (13), it remains to be determined if those indices also meet the value-validity condition in (14). This is considered next.

### Value validity (condition (14))

The Williams’ *E*_9_ meets the condition in (14) exactly since

19 $$ {E}_9=1-\frac{d\left({P}_S,{P}_S^1\right)}{d\left({P}_S^0,{P}_S^1\right)}=1-{\left(\frac{{\displaystyle \sum_{i=1}^S{\left({p}_i-1/S\right)}^2}}{{\left(1-1/S\right)}^2+\left(S-1\right){\left(1/S\right)}^2}\right)}^{1/2}=1-{\left(\frac{S{\displaystyle \sum_{i=1}^S{p}_i^2-1}}{S-1}\right)}^{1/2} $$

which equals *E*_9_ in Table 1. The *E*_9_ is also equivalent to the *coefficient of nominal variation* (*CNV*) proposed by Kvålseth (1995) as a measure of variation for nominal categorical data. It can also be expressed as a linear function of the standard deviation *s*_*S*_ of *p*_1_, …, *p*_*S*_ (with devisor *S*), i.e.,

$$ {E}_9=CNV=1-\frac{S}{\sqrt{S-1}}{s}_S $$

This is so rather obvious an evenness index that undoubtedly others have also thought of it.

The determination of whether or not Solomon’s *E*_10_ and this author’s *E*_11_ comply with the requirement in (14) then becomes a comparison of *E*_10_ and *E*_11_ with the ‘gold standard’ *E*_9_ for different distributions *P*_*S*_ = (*p*_1_, …, *p*_*S*_). The *E*_11_ is presented as an interesting alternative to *E*_10_, both of which are based on the ordering *p*_1_ ≥ *p*_2_ ≥ … ≥ *p*_*S*_. The *E*_11_ is based on the complement of the homogeneity (dominance, concentration) measure ${\displaystyle \sum_{i=1}^S{p}_i/i}$ proposed by Kvålseth (1993) and normed to the [0, 1] -interval. For large *S*, the denominator of *E*_11_ can more conveniently be computed as 1 − (*logS* + 0.5772)/*S* + 1/2*S*^2^, which is found to be accurate to four decimal places when *S* > 11 (see, e.g., Knopp 1990, p. 538).

In order to obtain the necessary data to determine if *E*_10_ and *E*_11_ comply with the requirement in (14), i.e., determine the extent to which *E*_10_ and *E*_11_ approximate *E*_9_, randomly generated distributions *P*_*S*_ = (*p*_1_, …, *p*_*S*_) were used. For each such *P*_*S*_, the value of *S* was first generated as a random integer between 2 and 30 (inclusive) and then each *p*_*i*_ was generated in descending order (*p*_1_ ≥ *p*_2_ ≥ … ≥ *p*_*S*_) as random numbers (to 6 decimal places) within the following intervals:

$$ \begin{array}{c}\hfill \frac{1}{S}\le {p}_1\le 1\hfill \\ {}\hfill \frac{1-{p}_1}{S-1}\le {p}_2\le \min \kern0.5em \left\{{p}_1,1-{p}_1\right\}\hfill \\ {}\hfill \vdots \hfill \\ {}\hfill \frac{1-{\displaystyle \sum_{j=1}^{S-2}{p}_j}}{S-\left(S-2\right)}\le {p}_{S-1}\le \min \left\{{p}_{S-2},\kern1em 1-{\displaystyle \sum_{j=1}^{S-2}{p}_j}\right\}\hfill \\ {}\hfill {p}_S=1-{\displaystyle \sum_{j=1}^{S-1}{p}_j}\hfill \end{array} $$

For each of the 25 such computer-generated distributions *P*_*S*_, the values of *E*_9_, *E*_10_, and *E*_11_ were computed as were the values of Pielou’s *E*_1_ in Table 1. Some (five) generated data sets were excluded since the index values were all nearly equal to 0 or 1. The normalized entropy *E*_1_ was included, although it clearly violates (13), since this entropy-based index appears to be the most popular evenness index. The results are summarized in Table 3.

**Table 3:** **Values of indices** *E*_1_, *E*_9_, *E*_10_, **and** *E*_11_ **defined in Table** 1 **for species distributions** *P*_*S*_ = (*p*_1_, …, *p*_*S*_) **with randomly generated** *S* **and** *p*_*i*_(*i* = 1, …, *S*)

**Data set**	***S***	***E***_**9**_	***E***_**10**_	***E***_**11**_	***E***_**1**_
1	15	0.27	0.22	0.25	0.44
2	7	0.88	0.81	0.87	0.98
3	18	0.45	0.24	0.44	0.53
4	2	0.15	0.15	0.15	0.39
5	4	0.29	0.22	0.26	0.51
6	12	0.35	0.11	0.28	0.42
7	17	0.92	0.91	0.92	0.99
8	14	0.32	0.30	0.31	0.51
9	8	0.85	0.77	0.84	0.97
10	17	0.63	0.56	0.61	0.82
11	29	0.84	0.84	0.84	0.95
12	5	0.19	0.10	0.15	0.33
13	30	0.38	0.30	0.35	0.53
14	5	0.32	0.18	0.28	0.49
15	27	0.82	0.69	0.79	0.92
16	2	0.29	0.29	0.29	0.59
17	5	0.52	0.40	0.50	0.74
18	30	0.31	0.15	0.24	0.39
19	10	0.57	0.42	0.53	0.75
20	26	0.95	0.91	0.94	0.99
21	19	0.67	0.61	0.66	0.84
22	19	0.77	0.76	0.76	0.91
23	17	0.46	0.40	0.44	0.65
24	12	0.74	0.70	0.73	0.91
25	2	0.50	0.50	0.50	0.81

As expected from the results in Table 2 together with (10), Table 3 shows that *E*_1_ substantially and consistently violates the value-validity condition in (14) since its values generally differ considerably from those of the ‘gold standard’ *E*_9_. The overstatement (positive value bias) of evenness by Pielou’s *E*_1_ appears to be as large as up to about 150 percent. By comparison, the approximation in (14) is much better for *E*_10_ and *E*_11_, i.e., the values of *E*_10_ and *E*_11_ in Table 3 are generally much closer to the corresponding values of *E*_9_ than are those of *E*_1_. This is especially the case for *E*_11_.

In the case of Solomon’s *E*_10_, the data in Table 3 indicate that values of *E*_10_ tend to be systematically and sometimes considerably smaller than those of *E*_9_. For data Sets 3, 6, 12, 14, and 18, the *E*_10_ values are half or less of the *E*_9_ values. If *E*_9_ is used to predict *E*_10_ as *Ê*_10_ = *E*_9_, then the coefficient of determination (when properly computed; see Kvålseth 1985) is found from Table 3 to be *R*^2^ = 1 − ∑(*E*_10_ − *E*_9_)^2^/∑(*E*_10_ − *Ē*_10_)^2^ = 0.86. Similarly, the root mean square difference (RMSD) between the values of *E*_10_ and *E*_9_ is found to be 0.10. When similarly comparing *E*_11_ with *E*_9_, it turns out that *R*^2^ = 0.99 and RMSD (*E*_11_, *E*_9_) = 0.03. That is, nearly 100% of the total variation of *E*_11_ (about its mean) is explained by the ‘fitted’ model *Ê*_11_ = *E*_9_ as compared to 86% in the case of *E*_10_. Consequently, *E*_11_ cannot be rejected for lacking value validity since it complies with the requirement in (13) and seems to have a high degree of approximation to *E*_9_ as required by (14). However, in the case of Solomon’s *E*_10_, while it complies with (13), one could certainly question its value validity since its approximation to *E*_9_ is rather marginal.

## Concluding comments

As advocated in this paper and about which most researchers seem to agree, an evenness index *E* should take on values over the interval from 0 to 1, with $E\left({P}_S^0\right)=0$ and $E\left({P}_S^1\right)=1$ for the distributions in (2). These fixed bounds are preferable when comparing the evenness of different species collections or communities with differing species richness *S*. Some indices have been proposed, however, for which the bounds depend on *S* such as $E\left({P}_S^0\right)=1/S$ for indices *E*_*T*_ in (16) by Taillie (1979) and *E*_*C*_ by Camargo (1993) defined as

$$ {E}_C=1-\frac{{\displaystyle \sum_{i=1}^{S-1}{\displaystyle \sum_{j=i+1}^S\left|{p}_i-{p}_j\right|}}}{S} $$

The *E*_*T*_ is seen to be a simple transformation of *E*_10_ in Table 1, i.e., *E*_*T*_ = [(*S* − 1)*E*_10_ + 1]/*S*. The *E*_*C*_ index has been referred to as Camargo’s evenness index (e.g., Magurran 2004, p. 118) although it is the same as the *E*_*T*_ proposed earlier by Taillie (1979). The fact that *E*_*C*_ = *E*_*T*_ follows directly from the equality

$$ {\displaystyle \sum_{i=1}^{S-1}{\displaystyle \sum_{j=i+1}^S\left|{p}_i-{p}_j\right|}}={\displaystyle \sum_{i=1}^S\left(S+1-2i\right){p}_i} $$

where *p*_1_, …, *p*_*S*_ on the right-hand side are ordered as in (3).

Since all the indices in Table 1 can be seen to have Properties P1-P4, with the exception of Alatalo’s *E*_5_ and Bulla’s *E*_8_ that are not strictly Schur-concave (Property P4), those indices can reasonably be expected to provide valid size (order) comparisons as in (7a). That is, for an evenness index *E* with Properties P1-P4 and for which, say, *E*(*P*_*S*_) = 0.90 and *E*(*Q*_*R*_) = 0.60, one can reasonably say that the species with abundance distribution *P*_*S*_ has higher evenness than that with abundance distribution *Q*_*R*_, but nothing more can be said with validity. One cannot credibly say that one has “considerably higher” evenness than the other or that the 0.90 value shows a “very high” degree of evenness even though the 0.90 value is near the top end of the [0, 1] -interval over which *E* is defined. Such interpretations and the types of difference comparisons in (7b)-(7c) require that *E* meets conditions (13)-(14) for value validity, conditions which are only clearly satisfied by Williams’ *E*_9_ and the new index *E*_11_ in Table 1.

Of the two indices *E*_9_ and *E*_11_ with the Properties P1-P5, there seems to be no particular reason for preferring *E*_11_ over *E*_9_. The *E*_9_ also has an intuitively appealing geometric interpretation in terms of the Euclidean distances between points in S-dimensional space in (19), i.e., *E*_9_ is the relative extent to which the distance between ${P}_S^1$ in (2) and the species abundance distribution (point) *P*_*S*_ = (*p*_1_, …, *p*_*S*_) is less than the distance between ${P}_S^1$ and ${P}_S^0$ defined in (2). Alternatively, since *P*_*S*_ is majorized by ${P}_S^0$ and since the distance $d\left({P}_S,{P}_S^1\right)$ can be shown to be strictly Schur-convex, the *E*_9_ in (19) can be expressed as

20 $$ {E}_9=1-\frac{d\left({P}_S,{P}_S^1\right)}{{\displaystyle \underset{P_S}{max}}\kern.2em d\left({P}_S,{P}_S^1\right)} $$

that is, *E*_9_ is the relative extent to which the distance between an observed *P*_*S*_ and the ${P}_S^1$ is less than the maximum such distance over all *S* -species distributions. For instance, from the data by Magurran (2004), pp. 226–227 of the abundances of 16 species of dung beetles around Bangalore, India, (19) or (20) gives *E*_9_(*P*_16_) = 0.48, which means that the distance between the dung beetle abundance distributions *P*_16_ and ${P}_{16}^1$ is 48% less than its maximum possible value for 16-species distributions. If, for these data with *S* = 16 and total number of dung beetles *N* = 1745, one uses ${P}_{16}^{0+}=\left(1730/1745,\ 1/1745,\dots,\ 1/1745\right)$ and ${P}_{16}^{1-}=\left(110/1745,\ 109/1745,\dots, 109/1745\right)$ instead of the ${P}_{16}^0$ and ${P}_{16}^1$ in (2) as discussed above, one obtains the distances $d\left({P}_{16},\ {P}_{16}^{1-}\right)=0.5034$ and $d\left({P}_{16}^{0+},\ {P}_{16}^{1-}\right)=0.9588$ so that, from (19), *E*_9_(*P*_16_) = 1 − 0.5034/0.9588 = 0.47.

In order to avoid misrepresentations and incorrect interpretations by using evenness indices lacking important properties, the conclusion from the analysis in this paper seems clear: *E*_9_ is the index of choice. Having all of the necessary properties (Properties P1-P5), *E*_9_ is a most informative index for which all of the comparisons in (7a)-(7c) can reasonably be considered valid.

## Appendices

### Appendix A

In order to prove that Bulla’s *E*_8_ in Table 1 is Schur-concave, but not strictly Schur-concave, consider the function

$$ {F}_{\alpha }={{\displaystyle \sum_{i=1}^S\left(\frac{p_i^{\alpha }+{\left(1/S\right)}^{\alpha }}{2}\right)}}^{1/\alpha }={\displaystyle \sum_{i=1}^S{m}_{\alpha i}} $$

where *α* is some arbitrary parameter. This *F*_*α*_ is simply the sum of arithmetic means *m*_*αi*_ of order *α* and of which *E*_8_ is the particular limiting member *F*_− ∞_ as *α* goes to − ∞. By taking the partial derivatives of *m*_*αi*_ with respect to *p*_*i*_, it is found that

$$ \frac{\partial^2{m}_{\alpha i}}{\partial {p}_i^2}=\left(\frac{\alpha -1}{4}\right){m}_{\alpha i}^{1-2\alpha }{\left(\frac{1}{S}\right)}^{\alpha }{p}_i^{\alpha -2} $$

which is negative for *α* < 1 so that *m*_*αi*_ is a strictly concave function of *p*_*i*_ for *α* < 1 and fixed *S*. Thus, being a sum of strictly concave functions, *F*_*α*_ is strictly Schur-concave for *α* < 1 (Marshall et al. 2011, p. 92). However, in the limit as *α* → − ∞, when (*F*_*α*_ − 1/*S*)/(1 − 1/*S*) becomes Bulla’s *E*_8_, the strict Schur-concavity does not hold as demonstrated by the earlier counterexample.

### Appendix B

As a proof of the parameter restrictions on Hill’s family of evenness indices in (17), consider the partial derivative of ${N}_{\alpha}^{\bullet }= log{N}_{\alpha }$ (which is the entropy family of Rényi 1961) with respect to *p*_*i*_, i.e.,

$$ \frac{\partial {N}_{\alpha}^{\bullet }}{\partial {p}_i}=\left(\frac{\alpha }{1-\alpha}\right){\left({\displaystyle \sum_{j=1}^S{p}_j^{\alpha }}\right)}^{-1}{p}_i^{\alpha -1} $$

which shows that, if *p*_1_, …, *p*_*S*_ are ordered as in (3), $\partial {N}_{\alpha}^{\bullet }/\partial {p}_i$ is strictly increasing in *i* = 1, …, *S* (when *p*_*i*_ > *p*_*i* + 1_ for all *i*) for *α* > 0 and strictly decreasing if *α* < 0. Therefore, ${N}_{\alpha}^{\bullet }$ and hence *N*_*α*_ are strictly Schur-concave for *α* > 0 and strictly Schur-convex for *α* < 0 (Marshall et al. 2011, pp. 84, 89). Consequently, since the ratio of a strictly Schur-concave function to a strictly Schur-convex function is strictly Schur-concave (Marshall et al. 2011, pp. 89, 145), the *E*_*Hαβ*_ in (17) is strictly Schur-concave for *α* > 0 and *β* < 0. It can also be proved that this result holds when either *α* = 0 or *β* = 0.
